# CD8+ Lymphocytes Control Viral Replication in SIVmac239-Infected Rhesus Macaques without Decreasing the Lifespan of Productively Infected Cells

**DOI:** 10.1371/journal.ppat.1000747

**Published:** 2010-01-29

**Authors:** Nichole R. Klatt, Emi Shudo, Alex M. Ortiz, Jessica C. Engram, Mirko Paiardini, Benton Lawson, Michael D. Miller, James Else, Ivona Pandrea, Jacob D. Estes, Cristian Apetrei, Joern E. Schmitz, Ruy M. Ribeiro, Alan S. Perelson, Guido Silvestri

**Affiliations:** 1 Department of Pathology and Laboratory Medicine, University of Pennsylvania, Philadelphia, Pennsylvania, United States of America; 2 Yerkes National Primate Research Center, Emory University, Atlanta, Georgia, United States of America; 3 Theoretical Biology and Biophysics, Los Alamos National Laboratory, Los Alamos, New Mexico, United States of America; 4 Gilead Sciences, Inc., Foster City, California, United States of America; 5 Tulane National Primate Research Center and Tulane Health Sciences Center, Tulane University, New Orleans, Louisiana, United States of America; 6 AIDS and Cancer Virus Program, Science Applications International Corporation-Frederick, Inc., National Cancer Institute, Frederick, Maryland, United States of America; 7 Beth Israel Deaconess Medical Center, Harvard Medical School, Boston, Massachusetts, United States of America; NIH/NIAID, United States of America

## Abstract

While CD8+ T cells are clearly important in controlling virus replication during HIV and SIV infections, the mechanisms underlying this antiviral effect remain poorly understood. In this study, we assessed the *in vivo* effect of CD8+ lymphocyte depletion on the lifespan of productively infected cells during chronic SIVmac239 infection of rhesus macaques. We treated two groups of animals that were either CD8+ lymphocyte-depleted or controls with antiretroviral therapy, and used mathematical modeling to assess the lifespan of infected cells either in the presence or absence of CD8+ lymphocytes. We found that, in both early (day 57 post-SIV) and late (day 177 post-SIV) chronic SIV infection, depletion of CD8+ lymphocytes did not result in a measurable increase in the lifespan of either short- or long-lived productively infected cells *in vivo*. This result indicates that the presence of CD8+ lymphocytes does not result in a noticeably shorter lifespan of productively SIV-infected cells, and thus that direct cell killing is unlikely to be the main mechanism underlying the antiviral effect of CD8+ T cells in SIV-infected macaques with high virus replication.

## Author Summary

Despite overwhelming evidence that CD8+ T cells are important in controlling virus replication during HIV and simian immunodeficiency virus (SIV) infections, the mechanisms responsible for this antiviral effect *in vivo* remain poorly understood. This lack of knowledge represents a key obstacle to our efforts to develop a CD8+ T cell-based AIDS vaccine. In this study, we implemented a new experimental system in which we determined the lifespan of productively SIV-infected cells *in vivo*, either in the presence or absence of CD8+ lymphocytes. The lifespan of productively infected cells was calculated based on the slope of the decline of SIV plasma viremia after initiation of ART using a widely accepted mathematical model. Using this novel approach, we determined that CD8+ lymphocytes control virus replication without noticeably decreasing the lifespan of productively infected cells, thus suggesting that the major mechanism of antiviral activity by CD8+ lymphocytes during pathogenic SIV infection may not be direct cell killing of productively SIV-infected cells.

## Introduction

The global spread of the HIV pandemic, currently affecting over 30 million individuals worldwide, emphasizes the urgency to develop a safe and effective vaccine. While many challenges face the AIDS vaccine development effort, the most fundamental obstacles are still at the level of the basic biology of the interaction between HIV and the human immune system [1]–[3]. These obstacles are: (i) the extreme heterogeneity of the virus; (ii) the lack of known correlates of immune protection against transmission or disease progression; (iii) the ability of the virus to become immunologically silent when the infection is latent; and (iv) the fact that any adaptive immune response to HIV or its non-human primate counterpart simian immunodeficiency virus (SIV) results in the generation of virus-specific, activated CD4+ T cells that are preferential targets for HIV and SIV. This latter effect may favor virus transmission and/or disease progression [1]–[3]. In this context, the disappointing results of the Merck STEP phase IIb clinical trial of a human adenovirus type 5 (AdHu5)-based candidate vaccine are just another indication of the tremendous challenge presented by these biological obstacles [4].

Due to the current absence of immunogens that can elicit HIV-specific neutralizing antibodies [5]–[7], numerous vaccine strategies have been proposed that are based on antiviral cellular immunity [8]. Virus-specific T cell responses, and, in particular, those mediated by CD8+ cytotoxic T lymphocytes (CTL) confer protection against many viral infections by favoring both viral clearance and resistance to re-infection [9],[10]. Several lines of evidence indicate that CD8+ T cells play an important role in anti-lentiviral immunity. First, CD8+ T cells can inhibit HIV and SIV replication in vitro [11],[12]. Second, there is a strong association between specific major histocompatibility alleles and rates of disease progression during HIV and SIV infection (reviewed in [13]). Third, CD8+ T cell escape mutants consistently arise during both acute and chronic HIV/SIV infections, indicating selective immune pressure on the virus population (reviewed in [14]). Fourth, there is a temporal association between post-peak decline of acute viremia and emergence of CD8+ T cell responses [15],[16]. While very informative, all these studies are correlative in nature and fail to establish a direct cause-effect relationship. The most convincing evidence for a direct antiviral effect of CD8+ T cells comes from a series of elegant studies demonstrating that antibody-mediated *in vivo* depletion of CD8+ lymphocytes is consistently associated with increased virus replication in SIV-infected rhesus macaques (RMs) [17]–[20]. Although this observation is very clear, the mechanisms by which CD8+ T cells exert anti-viral effects *in viv*o are still poorly understood. Conceivably, these mechanisms can be summarized into three major, non-mutually exclusive categories: CD8+ T cells may reduce production of virions by (i) direct killing of productively infected cells (thus decreasing their average lifespan); (ii) direct killing of infected cells before they begin producing virus, (iii) inhibition of the rate of virus production by non-cytolytic mechanisms; and (iv) reduction of the number of available target cells (i.e., activated CD4+ T cells) and hence the number of cells that become productively infected.

Elucidating the basis for the *in vivo* antiviral effect of CD8+ T cells will be important in designing of an effective, CD8+ T cell-based AIDS vaccine. In this study, our goal was to assess the relative contribution of cytotoxic T lymphocyte (CTL) activity to the antiviral effect of CD8+ lymphocytes. As previously proposed in [21], we reasoned that CD8+ T cell-mediated CTL activity will result in reduced production of virions per infected cell due to a significant shortening of the average *in vivo* lifespan of productively SIV-infected cells. In order to directly measure the impact of CD8+ lymphocytes on the lifespan of productively infected cells, we treated two groups of chronically SIVmac239-infected RMs with antiretroviral therapy (PMPA and FTC) in the absence or presence of CD8+ lymphocytes. We next calculated the lifespan of productively infected cells based on the slope of the decline of SIV plasma viremia after initiation of ART using a mathematical model [22]. We found that, during chronic SIVmac239 infection of RMs, depletion of CD8+ lymphocytes did not result in a significantly prolonged lifespan of infected cells *in vivo*. This result suggests that the CD8+ lymphocyte-mediated, direct killing of cells producing virus that results in shorter lifespan of these cells is unlikely to be the main mechanism underlying the antiviral effect of CD8+ T cells in SIV-infected macaques.

## Results

### Experimental design

**Figure 1:**
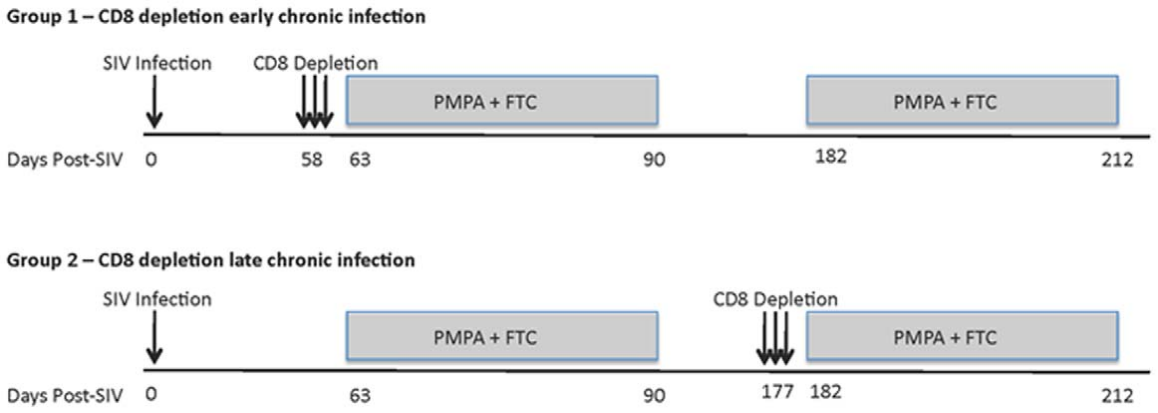
Experimental model to assess the lifespan of productively infected cells in the presence or absence of CD8+ T cells. Top panel, group A; CD8+ lymphocyte depletion and ART during early chronic phase, ART alone during late chronic phase. Bottom panel, group B; ART alone during early chronic phase, CD8+ lymphocyte depletion and ART during late chronic phase. Animals were given OKT8F (CD8-depleting mAb) for 3 consecutive days (Group A, days 58–60; Group B, days 177–179). Antiretretroviral therapy (PMPA and FTC) was given for 28 consecutive days.

In this study, we sought to better understand the mechanisms underlying the *in vivo* antiviral role of CD8+ lymphocytes during SIVmac239 infection of rhesus macaques (RM) by measuring the lifespan of productively infected cells in the presence or absence of CD8+ cells. To this end, we first infected ten RMs with 3,000 TCID50 of SIVmac239 and observed them throughout the acute phase of infection (peak and post-peak decline of viral load). We subsequently divided these ten SIVmac239-infected animals in two groups of five and treated them with potent antiretroviral therapy (ART) either alone (control animals) or after depletion of CD8+ lymphocytes with the OKT8F mAb (Figure 1). Several previous studies have demonstrated that analysis of changes in viral load after initiation of ART provides substantial insight into the dynamics of HIV and SIV infection [22]–[30]. Since reverse transcriptase inhibitors efficiently block de novo infections while not affecting productively infected cells, essentially all measurable virus originates from cells that were infected prior to treatment. As these cells die, measurable plasma viral loads decrease, and mathematical modeling can be used to determine the lifespan (or death-rate) of productively infected cells in vivo based on the rate of viral decay [22]. We applied this experimental/modeling approach and used two potent reverse transcriptase inhibitors, 9-R-(2-phosphono-methoxypropyl)adenine (PMPA) and beta-2′,3′-dideoxy-3′-thia-5-fluorocytidine (FTC) immediately after CD8+ lymphocyte depletion (or alone in the control animals). All RMs were given a cycle of ART for 28 days in two occasions: during early and late chronic infection. In the early chronic phase of infection (day 57) group A RMs (n = 5) were depleted of CD8+ lymphocytes immediately prior to treatment with ART, while group B animals (control, n = 5) were treated with ART alone. Conversely, during the late chronic phase of infection (day 177), group B animals (n = 4) were CD8+ lymphocyte depleted prior to ART, while group A animals (control, n = 3) was treated with ART alone. Note that the reduction in the number of animals per group during the late phase of our experiment was due to the fact that two RMs in group A and one in group B were sacrificed after the first cycle of ART because of severe weight loss and possible signs of simian AIDS. In all cases, the lifespan of productively infected cells *in vivo* (in the presence and absence of CD8+ T cells) was estimated using a mathematical model that allows the calculation of the lifespan of short and long-lived productively infected cells [22]. In addition, we analyzed the viral load decline data with a linear mixed effects model, where only the slope of the first phase of viral decline was estimated directly by a linear regression procedure.

### Treatment with OKT8F results in increased plasma viremia

**Figure 2:**
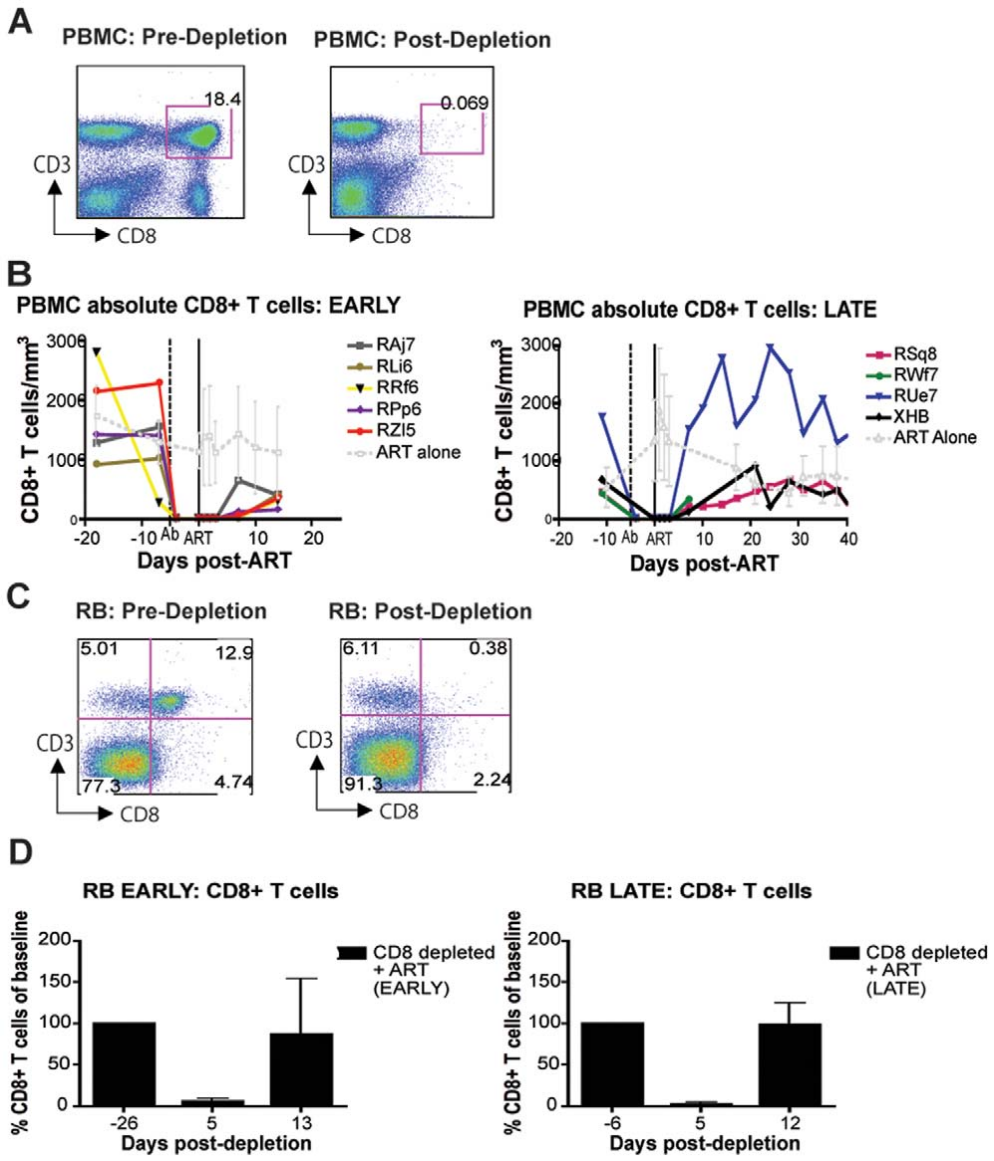
Administration of OKT8F results in near complete depletion of CD8+ lymphocytes. (**A**) Representative flow cytometry plots (x-axis, CD8; y-axis, CD3) demonstrating CD8+ lymphocyte levels in blood (left, 7 days before depletion; right, 6 days after depletion). (**B**) Longitudinal assessment of the absolute number of CD8+ T cells in peripheral blood for each animal during early chronic phase (left) or late chronic phase (right). Each colored line indicates an individual animal (CD8+ lymphocyte-depleted). Gray lines indicate the average CD8+ T cell number in non-depleted (ART alone) RMs. Dotted vertical line indicates the first day of depleting Ab treatment, solid vertical line indicates the first day of ART. (**C**) Representative flow cytometry plots (x-axis, CD8; y-axis, CD3) demonstrating CD8+ lymphocyte depletion in rectal biopsies (left, 10 days before depletion; right, 6 days after depletion). (**D**) Longitudinal assessment of the percent of CD8+ T cells (compared to baseline) in rectal biopsies during early chronic phase (left) or late chronic phase (right). Bars represent average of treated animals. CD8+ T cells previously gated on live lymphocytes.

**Figure 3:**
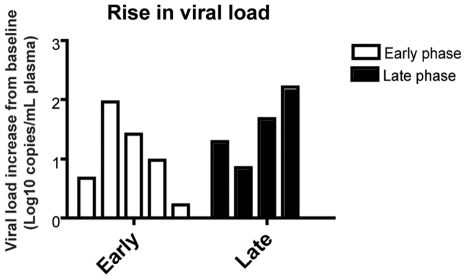
CD8+ lymphocyte depletion results in a 0.7–2.2 log_10_ rise in viral load. Change of viral load from baseline for each individual animal after CD8 depletion, during early chronic phase (white bars, left) or late chronic phase (black bars, right).

As expected based on our previous experience with the use of the OKT8F monoclonal antibody [31] (Engram, J. C. and Silvestri G., unpublished observations), all RMs treated with this antibody showed a very rapid and near complete depletion of CD8+ lymphocytes from both peripheral and mucosal tissues. During the first phase of this experiment (early chronic infection, i.e., day 57 post-inoculation), in group A animals, CD8+ T cells were depleted by an average of 99.97% (±0.01 s.d.) in peripheral blood (Figure 2A, 2B), 99.29% (±0.50 s.d.) in rectal biopsies (Figure 2C, 2D), and 99.23% (±1.07 s.d.) in bronchoalveolar lavage (data not shown) as measured by flow cytometry. During the second phase of this experiment (late chronic infection, i.e., day 177 post inoculation), in group B animals, CD8+ T cells were depleted by an average of 99.95% (±0.03 s.d.) in peripheral blood (Figure 2B) and 98.07% (±1.54 s.d.) in rectal biopsies (Figure 2D) as measured by flow cytometry. Extent of depletion in mucosal tissues was corrected for non-CD8+ T cell fluctuations (as described in [32]). In all cases, and consistent with previous studies [18], CD8+ T cells were depleted very rapidly (>98% depletion after 24 hours) and CD8+ T cell depletion was sustained for 8–13 days with nadir depletion occurring between 5-6 days after the first infusion. Of note, the OKT8F Ab induced a loss of both CD3+CD8+ cells as well as CD3−CD8+ cells, thus indicating that not only CD8+ T cells, but also NK cells, NKT cells, and TCRγδ T cells that express CD8 are also efficiently depleted in this experimental setting. Similar to previous studies in which CD8+ T cells were depleted during pathogenic SIV infection [17],[18],[19],[20], we observed an increase in viremia between 0.7–2.2 logs (Figure 3).

### Suppression of virus replication after treatment with PMPA and FTC

**Figure 4:**
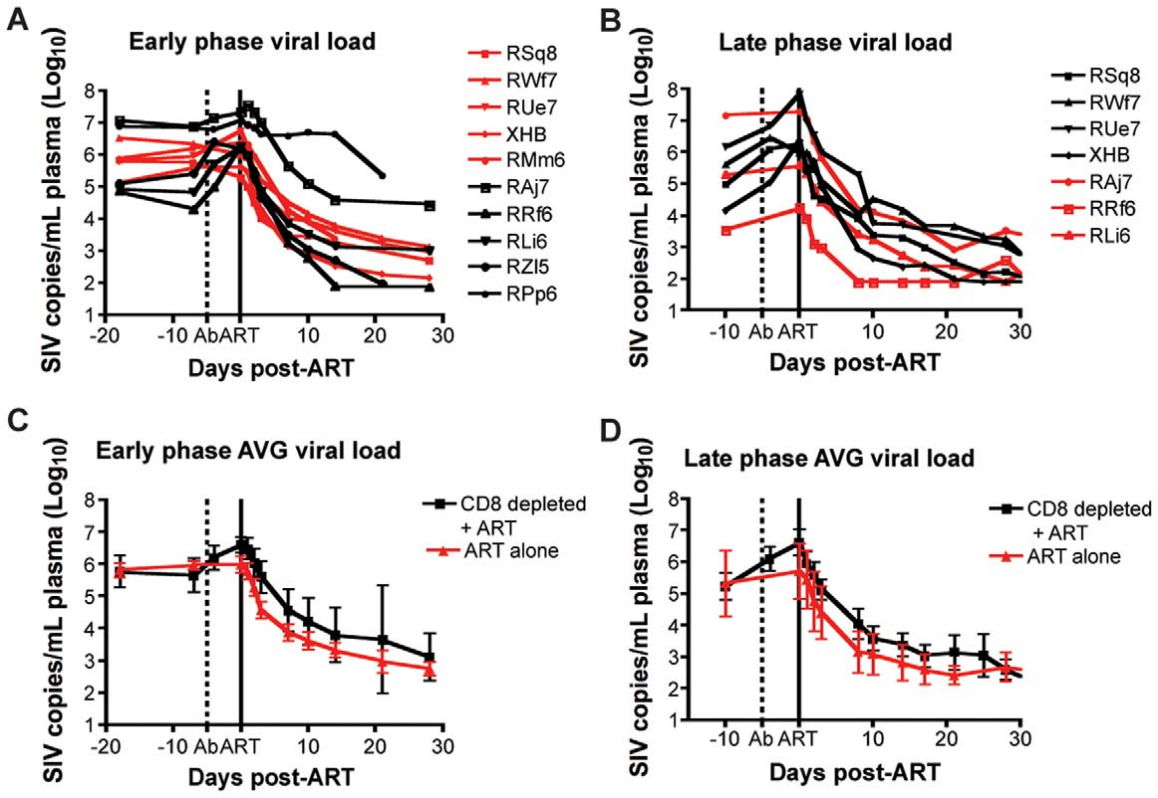
Treatment with PMPA and FTC effectively suppresses virus replication in SIVmac239-infected RMs. (**A**, **B**) Plasma viral load (log_10_) measured longitudinally for each individual animal (black lines, CD8+ lymphocyte-depleted, red lines, control) during (**A**) early chronic phase or (**B**) late chronic phase. (**C**,**D**) Average plasma viral load (log_10_) for each group (black, CD8+ lymphocyte-depleted; red, control) during (**C**) early chronic phase or (**D**) late chronic phase. Error bars represent standard deviation. Dotted vertical line indicates the first day of depleting Ab treatment, solid vertical line indicates the first day of ART.

In this study, antiretroviral therapy with PMPA and FTC (30mg/kg/day i.m. for each drug, for a total of 28 days of treatment) was conducted during both early and late phases of the study (i.e., starting at days 63 and 182 post-SIV infection, respectively). In the CD8+ lymphocyte-depleted animals, this timing corresponded to initiation of ART three days after the last OKT8F infusion. As expected, during both phases of the study, ART effectively suppressed virus replication in all RMs by at least 0.5 log_10_ (and in 16 out of 17 instances of treatment by at least 1.5 log_10_) within a week after initiation of therapy (Figure 4). The observation that ART induced a rapid and dramatic suppression of SIV replication allowed us to proceed to the next phase of the study in which the kinetics of decline of plasma viremia were used to calculate the lifespan of cells producing virions *in vivo*.

### CD8+ lymphocyte depletion does not prolong the lifespan of SIV-infected cells in vivo

Previous studies [22]–[24],[26] demonstrated that there are two phases of viral decay; an initial rapid, exponential decline of 1–2 logs, in which productively infected short-lived cells are lost, followed by the second phase, which is characterized by a slower decline, where long-lived infected cells are lost. In order to quantify the contribution of CD8+ T cells to the lifespan of productively infected cells in vivo, we quantified this parameter in the presence or absence of CD8+ T cells by analyzing the viral decline after initiation of ART using the equation:
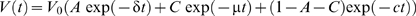
(1)where *A = (* *NkT* *_o_)/(c−δ)*, *C = (c−* *NkT* *_o_)/(c−μ)* and *V_0_* is the initial viral load, *k* is the infection rate, *N* is the viral burst size, δ is the death rate of short-lived productively infected cells, μ is the death rate of long-lived productively infected cells, and *c* is the rate of virion clearance [22] (Figure 4). By fitting the natural logarithm of *V(t)* given by equation *(1* *)* to the natural logarithm of the measured SIV RNA between initiation and termination of therapy, we were able to estimate *δ* and μ, the death-rate of short-lived and long-lived productively infected cells, for each animal either in the presence or absence of CD8+ T cells (Table S1). However, for macaques RAj7, RMm6 and RPp6 in early infection, we could not fit a second phase decline due to too few data points or a flat second phase. Thus for these animals we used a monophasic decline model [22],[24], appropriate for reverse transcription inhibitor therapy, by making *C* = 0 in (Eq. *1*), i.e., setting *NKT* *_0_ = c*, and estimated δ but not μ.

**Figure 5:**
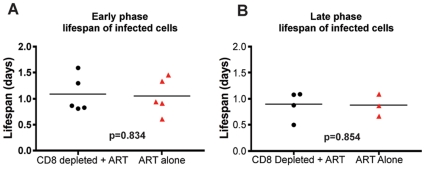
CD8+ lymphocyte depletion does not affect the lifespan of infected cells during SIV infection. The estimated lifespan of productively infected cells, 1/δ, for each animal; CD8+ lymphocyte-depleted (black) and control (red) during (**A**) early chronic phase or (**B**) late chronic phase. P = n.s.

During both the early and late phases of this study, we found that the lifespan of short-lived productively infected cells (1/*δ*) is similar regardless of the presence or absence of CD8+ T cells. Specifically, during the early phase, the mean lifespan for group A (CD8+ lymphocyte depleted RMs) was 1.11 (±0.39 s.d.) days (median = 0.87), while the mean lifespan for group B (control non-CD8+ lymphocyte depleted animals) was 1.05 (±0.35 s.d.) days (median = 0.93) (p = 0.83) (Figure 5A, Table S1). During the late phase, the mean lifespan for group A (control non-CD8+ lymphocyte depleted) was 0.87 (±0.21 s.d.) days (median = 0.86), while the mean lifespan for group B (CD8+ lymphocyte depleted) was 0.89 (±0.28 s.d.) days (median = 0.98) (p = 0.85) (Figure 5B, Table S1). These data indicate that the depletion of CD8+ lymphocytes does not prolong the lifespan of short-lived productively infected cells *in vivo* during pathogenic SIV infection of RMs. Similarly, the estimated lifespans of long-lived infected cells (1/µ) were also not different between CD8+ lymphocyte depleted and not depleted animals (9.7±10.7 vs. 8.5±5.6 days, respectively, p = 0.71). A notable observation, however, is that of the seven RMs that participated in both the early and late phase studies, five had a shorter lifespan of short-lived infected cells (by an average of 36%) later in infection. This finding, together with the slightly higher increase in viremia that we observed after CD8+ lymphocyte depletion in the late phase as compared to the early phase (Figure 3) is somewhat unexpected as CD8+ T cells are thought to undergo progressive exhaustion during chronic HIV and SIV infections [33],[34],[35],[36],[37],[38],[39], and suggests further how the mechanisms by which CD8+ T cells control virus replication is likely more complex than previously appreciated.

To further confirm these results, and avoid any potential bias from the modeling approach used, we also analyzed the observed first-phase decays of the logarithm of the viral load during treatment with a linear mixed-effects model. In this approach, we tested directly whether the slopes of the first-phase decay in the data are different in the two groups, with each animal as a random sample from treated or untreated macaque. Again, we did not find any differences in the slopes in either the acute or chronic groups (p = 0.58, and p = 0.81, respectively), thus lending support to our conclusions that depletion of CD8+ lymphocytes does not affect the dynamics of viral decay. We note that this approach with linear mixed effects models makes optimal use of the data by fitting a simple line to the decay and taking into consideration all the available data at the same time (all animals from both treatment groups).

A caveat to this analysis is that the mathematical model (Eq. *1*) used to determine the death-rate of infected cells is based on the assumption that the virus and the target cells are at their set-point or steady state levels upon the initiation of therapy and that therapy is 100% effective in blocking new infections [22]. However, CD8+ lymphocyte depletion causes two perturbations to the steady state: (i) an increase in viremia prior to ART treatment (Figure 3), and (ii) a potential increase in the level of activated CD4+ T cells, thus expanding the target cell population for virus replication. First, to determine the effect of changes in viremia after CD8+ lymphocyte depletion, surrogate data for SIV kinetics with virus not in steady state were created by equation *(2)* (in Text S1) with a known value of δ and then fit using equation *(1* *)* to assess if, and to what extent, viral load increases before the start of therapy altered estimated δ values (Text S1, Figure S1). Second, to take into account the possibility that significant changes in the activation state of CD4+ T cells occurs after CD8+ lymphocyte depletion, we created surrogate data that include changes in target cells (Text S2, Figure S2). Third, the above analyses were repeated with various drug effectiveness less than 100% to study the influence of this factor on our estimate of δ (Text S2). All three analyses demonstrated that errors due to lack of steady-state viremia, to changes in target cell pools after CD8+ lymphocyte depletion as well as to drug effectiveness <100% lead to a potential underestimation of both δ and μ (Text S1 and S2). Further, when the drug effectiveness was high, i.e. 99%, the maximum error in estimating δ and μ was <3.5%. This analysis shows that the actual values of δ and μ in systems with CD8+ lymphocyte depletion may be even higher than we estimate, thus supporting our conclusion that lack of CD8+ T cells does not increase the lifespan of productively infected cells.

A conceivable conceptual limitation of our experimental system is that antiretroviral treatment might have an immediate impact on the number and/or function of SIV-specific CD8+ T cells, thus introducing a potential bias in our effort to assess the impact of CTL activity on the lifespan of infected cells based on the decline of viremia after ART. To directly address this issue, we measured the magnitude and functionality of SIV-specific CD8+ T cells before and after ART in non-CD8+ lymphocyte depleted animals and found that ART did not cause any significant changes in SIV-specific CD8+ T cell responses during either the early or late phase of the study (data not shown), therefore not supporting the possibility that the use of ART generated an intrinsic bias in our assessment of the impact of CD8+ lymphocytes on the lifespan of SIV-infected cells.

### CD8+ lymphocyte depletion is associated with decreased plasma levels of chemokines and cytokines

**Figure 6:**
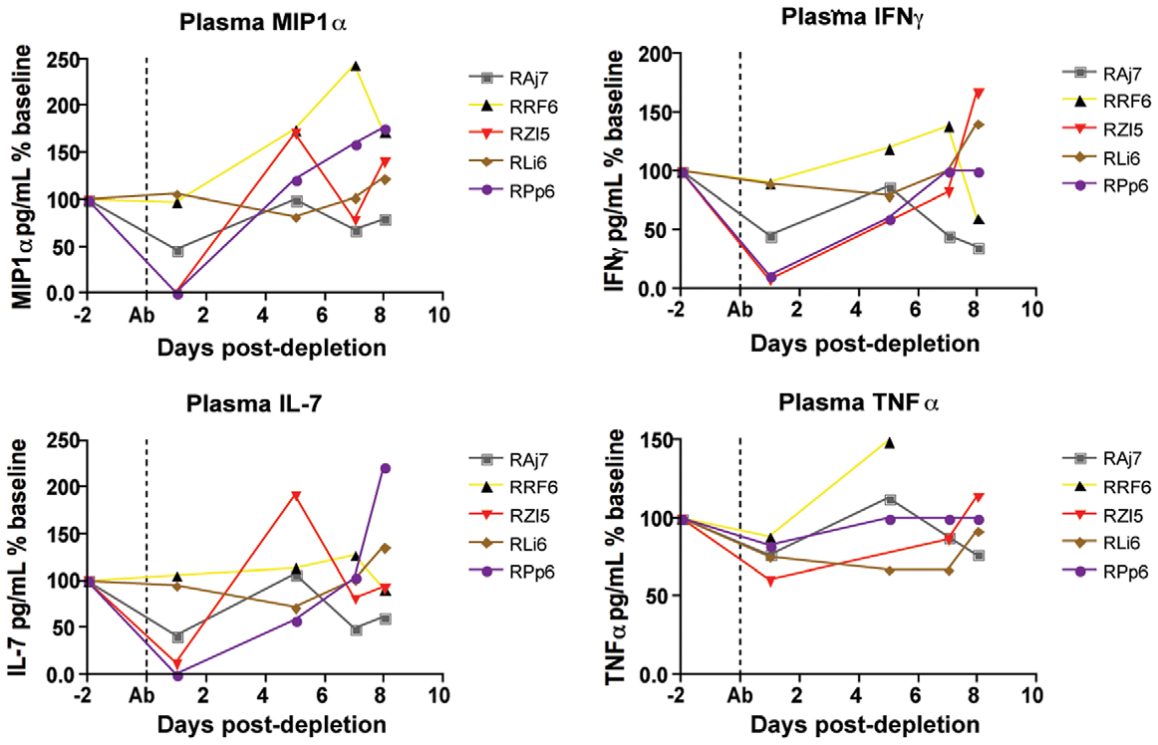
Changes in chemokines and cytokines after CD8+ lymphocyte depletion. Levels of plasma MIP1α (top left), IFNγ (top right), IL-7 (bottom left) and TNFα (bottom right) were measured in all animals after CD8+ lymphocyte depletion, early phase shown here. Dotted line indicates first day of depleting treatment.

As discussed above, the results of this study support the hypothesis that the strong antiviral effect of CD8+ lymphocytes during chronic SIVmac239 infection of RMs is due to mechanisms that do not affect the lifespan of productively infected cells. Potential non-cytolytic mechanisms of SIV suppression by CD8+ T cells include the block of virus spread and entry via production of chemokines such as CCL3/MIP-1α, CCL4/MIP-1β, and CCL5/RANTES). To address this possibility we measured the plasma levels of these chemokines and numerous other cytokines, including those with potential antiviral activity such as TNFα, IFN-α, and IFN-γ, in the plasma of the SIV-infected RMs included in this study at multiple time points after CD8+ lymphocyte depletion. In most instances, cytokine plasma levels were either unchanged or showed only irregular fluctuations after CD8 depletion, thus possibly reflecting the very local nature of many of these factors. As such, this result does not necessary rule out that changes in the concentration of certain cytokines may occur *in vivo* in specific anatomic microenvironments. However, as shown in Figure 6, we found that, in several animals, CD8+ lymphocytes depletion is followed by a dramatic decline in the plasma levels of MIP-1α, IFN-γ, IL-7 and TNFα. MIP-1α is a CCR5-binding chemokine which may directly compete with SIV *in vivo*, and whose plasma concentration was decreased after CD8+ lymphocyte depletion to an average of 50% (±51%) of baseline levels. Plasma levels of the pro-inflammatory and potentially antiviral cytokines IFN-γανδ TNFα were also, on average, reduced to 49% (±40%) and to 76% (±10%) of baseline levels, respectively, after CD8+ lymphocyte depletion. Plasma concentrations of the lympho-tropic cytokine IL-7 were also decreased to 51% (±48%) of baseline levels after CD8+ lymphocyte depletion. As all of these cytokines may have an important antiviral effect during SIV infection, lower levels of these molecules after CD8+ lymphocyte depletion may contribute to the observed rise in viremia. While these data are not conclusive, they suggest that soluble factors produced by CD8+ lymphocytes may play a key role in the suppression of virus replication mediated by these cells in SIV-infected RMs.

### Effects of CD8+ lymphocyte depletion on CD4+ T cell activation

**Figure 7:**
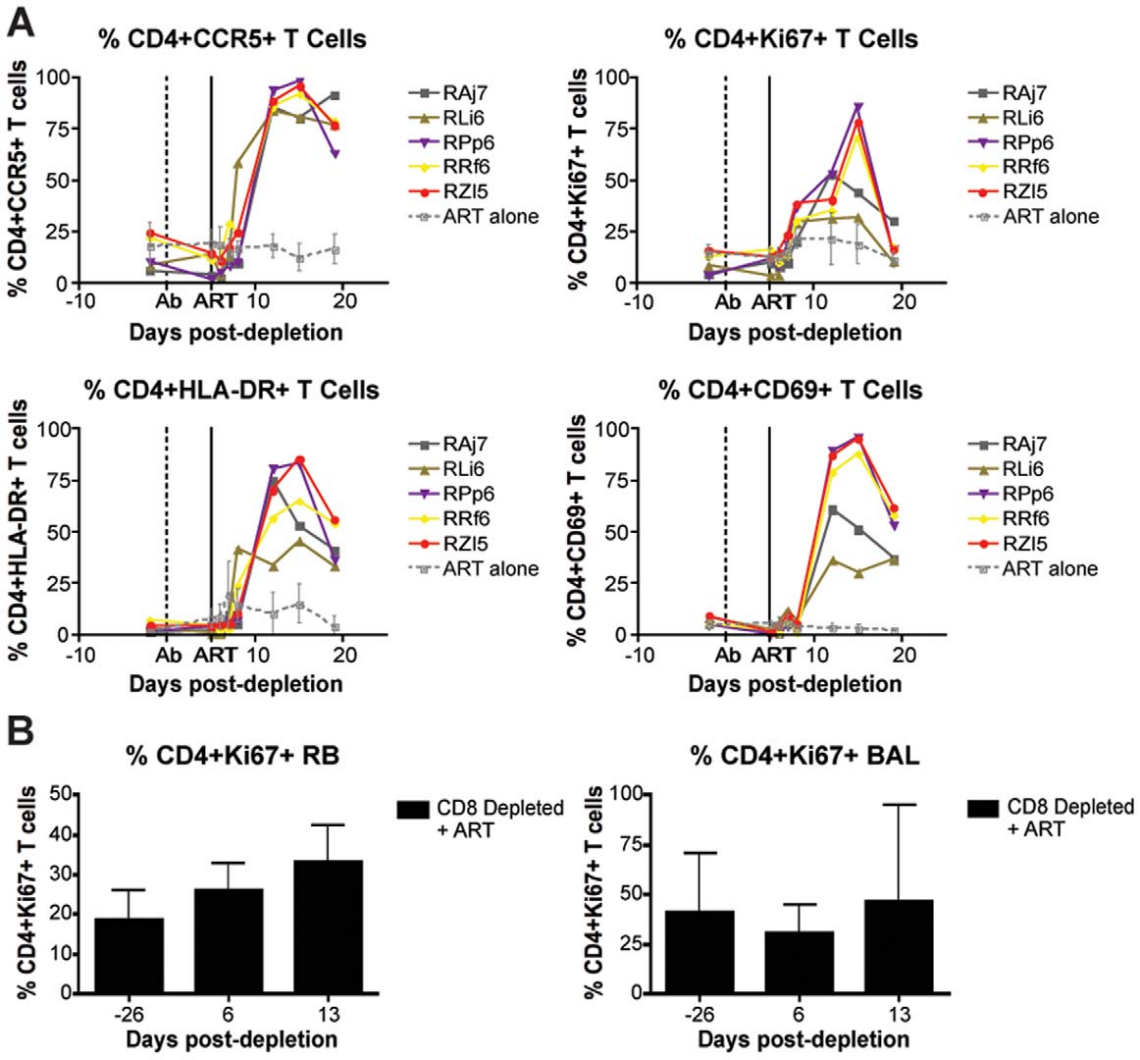
CD8+ lymphocyte depletion results in a rise in activated CD4+ T cells. (**A**) Longitudinal assessment (individual animals from CD8-depleted group and mean and s.d. from control group) of the percent of CD4+CCR5+ (top left), CD4+Ki67+ (top right), CD4+HLA-DR+ (bottom left), and CD4+CD69+ (bottom right) T cells during early chronic infection. (**B**) Longitudinal assessment of the mean (and s.d.) percent of CD4+Ki67+ T cells in rectal biopsies (left) and bronchoalveolar lavage (right).

The finding that CD8+ lymphocyte depletion does not result in a prolonged lifespan of productively infected cells is also consistent with the possibility that the observed increase in virus replication is caused, at least in part, by increased CD4+ T cell activation, which would result in an increased availability of target cells for SIV infection. Several factors may be involved in this CD4+ T cell activation, including homeostatic responses to lymphopenia, increased availability of CD4+ T cell tropic and/or pro-inflammatory cytokines, reactivation of latent virus infections, and other potential changes in the lymphoid microenvironment(s). To address this possibility, we measured the expression of activation and proliferation markers in CD4+ T cells before and after CD8+ lymphocyte depletion. As shown in Figure 7, we found that CD8+ lymphocyte depletion was followed by a marked increase in CD4+ T cell activation that occurred in all examined tissues. In peripheral blood, the peak of CD4+ T cell activation occurred at day 15 post-depletion, and most activation did not increase at all until day 8. On average at peak activation, the fraction of CD4+Ki67+ T cells was 6.7 fold higher than baseline levels, the fraction of CD4+CCR5+ T cells was 6.2 fold higher than baseline, the fraction of CD4+HLA-DR+ T cells was 19.2 fold higher than baseline, and the fraction of CD4+CD69+ T cells was 10.6 fold higher than baseline levels (Figure 7A). The kinetics of CD4+ T cell activation was also delayed in mucosal tissues, although it should be noted that the relative infrequent sampling schedule raises the possibility that we missed the peak of CD4+ T cell activation in these tissues. In rectal biopsies, during early chronic infection, the fraction of CD4+Ki67+ T cells was 1.4 fold higher than baseline levels at day 6 post-depletion and 1.8 fold higher than baseline levels at day 13 post-depletion (Figure 7B, left). Similarly, during late chronic infection, CD4+Ki67+ T cells were 0.9 fold higher than baseline at day 5 post-depletion, and 1.2 fold higher than baseline levels at day 12 post-depletion (data not shown). The same trend was observed in bronchoalveolar lavage, where CD4+Ki67+ T cells were 0.7 fold higher than baseline at day 6 post-depletion, and 1.5 fold higher at day 13 post-depletion (Figure 7B, right). Importantly, the observed changes in CD4+ T cell activation followed, rather than preceded, the increase in plasma viral load, thus suggesting that the activation of CD4+ T cells that occurs after CD8+ lymphocyte depletion is unlikely to be the predominant source of the increased viremia. As such, these data support a model in which CD8+ T cells play a key, direct role in maintaining the steady state of viral load during chronic SIV infection.

## Discussion

Numerous studies indicate that CD8+ lymphocytes play an important role in suppressing virus replication *in vivo* during pathogenic HIV and SIV infections [11], [13]–[17]. However, the mechanisms underlying this activity are still poorly understood and may involve several non-mutually exclusive factors, whose relative contribution to the net *in vivo* antiviral effect of CD8+ lymphocytes is unknown. Direct killing of productively HIV- or SIV-infected cells by CD8+ T cells (i.e., CTL activity) has been shown in many *in vitro* settings and is very likely to occur *in vivo* as well [40]. In addition, suppression of HIV replication by CD8+ T cells via non-cytolytic mechanisms that inhibit virus transcription was first observed by Levy and colleagues in 1986 [11],[41],[42], although the nature of this antiviral activity has not been fully elucidated [43]. Furthermore, CD8+ T cells may block HIV/SIV spread from cell-to-cell by releasing factors such as CCR5-binding chemokines (i.e., MIP-1a/CCL3, MIP-1b/CCL4, and RANTES/CCL5) that act as competitive inhibitors of CCR5-mediated virus entry [44],[45]. Finally, it is conceivable that the increased HIV/SIV replication observed after CD8+ lymphocyte depletion is caused, at least in part, by changes in the activation state of CD4+ T cells that render these cells more intrinsically “permissive” to virus replication [31].

In this study, we set to address the relative contribution of cytolytic vs. non-cytolytic mechanisms of CD8+ lymphocyte-mediated control of virus replication by measuring the *in vivo* lifespan of productively infected cells during chronic SIVmac239 infection of RMs in the presence or absence of CD8+ lymphocytes. The assessment of the turnover of infected cells was conducted using a well-characterized mathematical model that is based on the analysis of the decline of viral load after initiation of antiretroviral therapy [22]. Of note, the experimental design of this study (Figure 1) is based on the premise that SIV-specific CTL activity will ostensibly reduce virus replication by shortening the average *in vivo* lifespan of productively infected cells [21]. To the best of our knowledge, this is the first direct assessment of the impact of CD8+ T cells on the longevity of productively SIV-infected cells *in vivo*.

Perhaps surprisingly, the results of this experiment indicate that CD8+ T cells do not affect the lifespan of productively infected cells during SIVmac239 infection of rhesus macaques. Our experiments further confirmed the important role of CD8+ T cells in antiviral immunity since, in all circumstances, the *in vivo* depletion of CD8+ lymphocytes is associated with a marked and consistent increase in viral load (Figure 3). However, our experiments do challenge the common assumption that the main antiviral effect of CD8+ T cells is related to the direct killing of productively infected CD4+ T cells (i.e., CTL activity) that suppresses virus replication by reducing the amount of time in which infected cells are able to produce virions (Figure 5). Instead, these results indicate that non-cytolytic mechanisms of SIV inhibition are potentially involved, or that CD8+ T cells have cytolytic affects prior to productive virus replication.

While CD8+ T cell-mediated CTL activity may play a key role in killing infected cells before they start producing virus and/or in “elite controller” SIV-infected RMs with very low viremia, this study is consistent with a model wherein, during chronic SIVmac239 infection of RMs with high viremia, the main antiviral effect of CD8+ lymphocytes may be due to non-cytolytic mechanisms that do not impact the average lifespan of infected cells. These non-cytolytic mechanisms may include the inhibition of SIV production by factors acting at the level of virus transcriptions and/or the block of virus spread and entry via production of chemokines and cytokines. This latter possibility is supported by our observation that, in several animals, CD8+ lymphocyte depletion is followed by a dramatic decline of the plasma levels of molecules such as the CCR5-binding chemokine MIP-1α, the antiviral cytokine IFN-γ, the pro-inflammatory cytokine TNFα and the homeostatic cytokine IL-7 (Figure 6). The finding that the absence of CD8+ T cells does not noticeably increase the lifespan of productively infected cells is also consistent with the possibility that the increase in viral load after CD8+ lymphocyte depletion is caused by increased availability of activated CD4+ T cells as targets for virus replication. In this experiment, CD8+ lymphocyte depletion was indeed followed by a marked increase in CD4+ T cell activation that occurred in all examined tissues (Figure 7). However, the observed changes in CD4+ T cell activation followed, rather than preceded, the increase in viremia, thus suggesting that activation of CD4+ T cells after CD8+ lymphocyte depletion is unlikely to be the main mechanism for the increase in viremia. Further studies in which the level of CD4+ T cell activation following CD8+ lymphocyte depletion is examined at earlier time points and in more tissues may be needed to better assess how changes in CD4+ T cell activation may contribute to the increase in virus replication in SIV-infected, CD8+ lymphocyte depleted RMs.

One caveat of this study is that the OKT8F depleting Ab also depletes NK cells, NKT cells, and TCRγδ T cells that express the CD8 molecule. NK cells and NKT cells are known to have an antiviral role during HIV infection, including the production of proinflammatory cytokines and chemokines which drive a Th1 antiviral immune response [46]–[49]. TCRγδ T cells also may play a role in antiviral immunity during SIV/HIV infection, as these cells have a specific role in the recognition of microbial pathogens and produce both Th1 and Th2 cytokines that can influence the adaptive immune response after infection [50]. Therefore, loss of any of these cell types may influence viremia after CD8+ lymphocyte depletion. However, these considerations do not change the conclusion that removal of CD8+ lymphocytes does not affect the lifespan of cells productively infected with SIV. As mentioned above, an additional caveat to this study is that, while it is clear that CD8+ lymphocyte depletion does not prolong the lifespan of productively SIV-infected cells *in vivo*, it remains possible that CD8+ lymphocytes exert their antiviral effect by killing infected cells before they start producing new virions. In this case, this cytolytic antiviral effect would not translate into a net change of the average lifespan of productively infected cells. Further investigation will be required to quantify the *in vivo* impact of this putative antiviral effect, and to compare it with the impact of non-cytolytic mechanisms of SIV suppression.

Modeling has also suggested two other scenarios in which viral load perturbation by drug therapy might not be able to detect and quantify a cytolytic effect of CD8+ T cells [51]. The first possibility is that a fraction of SIV-infected cells that were not exposed to CTL-mediated killing become the major virus producers, thus making the viral decay slope independent of CD8+ lymphocyte-mediated killing. In other words, if the virus has escaped the CTL response, or is hidden from it, then CD8+ lymphocyte depletion will have little effect on the lifespan of productively infected cells. This scenario, however, does not explain why CD8+ lymphocyte depletion is consistently followed by a major increase in viral load. The second possibility is that the rate at which an infected cell becomes a target of CD8+ T cell-mediated killing is slow and represents the rate-limiting step in viral kinetics. In this case, the first-phase decay slope will reflect this rate and not the rate of killing of productively infected cells. [51]. However, this is a highly unlikely scenario as presentation of incoming, Gag-derived epitopes occurs as early as 2 hours post-infection [52], and the step of becoming a target should therefore not be rate-limiting.

The possibility that non-cytolytic mechanisms are central to the antiviral activity of CD8+ T cells *in vivo* is consistent with several previous observations. First, a prominent role for CD8+ lymphocyte-derived factors that can block virus entry is reflected by the known antiviral activity of CCR5-binding chemokines [44], as well as the protective effect of increased gene copies of CCL3L1 [53]. Second, a non-cytolytic antiviral effect of CD8+ T cells would be consistent with the classical observations that CD8+ T cells produce a soluble antiviral factor that suppresses HIV transcription (reviewed in [43]). Third, our results are in agreement with theoretical predictions on the *in vivo* role of CD8+ T cells that were made several years ago based on the kinetics of viral increase observed post CD8+ T cell depletion [18]. Of note, the notion of a predominance of non-cytolytic mechanisms of CD8+ T cell-mediated suppression of SIV replication does not necessarily conflict with the known observation that CTL escape variants are selected for *in vivo* during both HIV and SIV infections [14]. Indeed, it is possible that the presence of escape mutants results in a decreased or absent stimulation of SIV-specific CD8+ T cells which, in turn, creates a micro-environment more favorable to the production of virions. In this scenario, escaped mutants of SIV will be positively selected even in absence of an antiviral effect due to direct cytolytic activity.

In conclusion, this study demonstrated, for the first time, that during chronic SIV infection of RMs with high virus replication, the antiviral effect of CD8+ T cells is due to mechanisms that do not affect the *in vivo* longevity of productively infected cells. In our view, this result provides an important advance in our understanding of the correlates of CD8+ T cell-mediated protection from SIV replication, which may inform the rational design of AIDS vaccines whose efficacy relies on the antiviral effect of CD8+ lymphocytes.

## Materials and Methods

### Animals

Ten rhesus macaques of Indian origin (of which 6 were MamuA*01, equally distributed 3/group) were infected with 3000 TCID50 of SIVmac239 i.v. for this study. All animals were housed at the Yerkes National Primate Research Center and maintained in accordance with NIH guidelines. The number of RMs used for this study were determined based on power analysis (Text S3). RMs belonging to the two groups were age and weight matched. These studies were approved by the Emory University and University of Pennsylvania Institutional Animal Care and Use Committees.

### CD8+ lymphocyte depletion

RMs were treated with 4mg/kg/day i.v. of OKT8F mAb for three consecutive days. Depletion efficiency in blood was calculated based on flow cytometric analysis and complete blood cell counts; depletion efficiency in tissues other than blood (where absolute number calculations were not available) was calculated based on flow cytometry, as fraction of the baseline percent of CD8+ T cells.

### Antiretroviral therapy

Reverse transcriptase inhibitors 9-*R*-(2-phosphonomethoxypropyl)adenine (PMPA; tenofovir) and beta-2,3-dideoxy-3-thia-5-fluorocytidine (FTC; emtricitabine) were provided by Gilead Sciences and administered to each animal i.m. (30mg/kg/animal/day each) for 28 days during both early and late phases of the study (treatment began at days 63 and 182 post-SIV infection, respectively).

### Sample collection and processing

Peripheral blood mononuclear cells were isolated by gradient centrifugation (ficoll). Procedures for lymph node biopsies, rectal biopsies, and bronochoalveolar lavage as well as isolation of lymphocytes form the obtained samples were performed as previously described [32].

### Immunophenotyping and flow cytometry

Multicolor flow cytometric analysis was performed on whole blood or isolated cells according to standard procedures using human mAbs that crossreact with RMs. Predetermined optimal concentrations were used of the following antibodies: anti-CD3-Alexa700 (clone SP34-2, BDPharmigen), anti-CD8-PacOrange (clone RPA-T8, BDPharmigen), anti-CD8-PE-TR (clone RPA-T8, Caltag/Invitrogen), anti-CD4-PE-Cy5.5 (clone OKT4, eBioscience), anti-CD4-PerCP-Cy5.5 (clone L200, BDPharmigen), anti-CD4-PacBlue (clone OKT4, eBioscience), anti-Ki67-FITC (clone B26, BDPharmigen), anti-CCR5-PE (clone 3A9, BDPharmigen), anti-CD69-PE-Cy7 (clone FN50, BDPharmigen), anti-HLA-DR-PE-Cy5 (clone L243, BDPharmigen), Aqua Live/Dead amine dye-AmCyan (Invitrogen). All samples were permeabilized and fixed using CytoFix/Perm Kit (BDPharmigen) and intracellularly stained to detect Ki67. Flow cytometric acquisition was performed on at least 100,000 lymphocytes on an LSRII cytometer driven by the FACS DiVa software (version 5.2; BD). Analysis of the acquired data was performed using FlowJo software (version 8.7.1; TreeStar). For all analysis of specific cell subsets, we used a threshold of 200 collected events.

### Plasma viral loads

Quantitative real-time reverse-transcriptase (RT)-PCR assay to determine SIVmac239 plasma viremia was performed as previously described [54].

### SIV-specific T cell responses

SIV-specific T cell responses were measured by intracellular cytokine staining for interferon-γ (IFN-γ), tumor necrosis factor-α (TNF-α), and Interleukin-2 (IL-2), as well as the degranulation marker CD107a, in response to pools of 15-mer peptides overlapping by 11 amino acids and spanning the entire sequence of three major antigenic proteins of SIVmac239 (gag, pol, env) as detailed in [55] and using the following antibodies: anti-human IFN-γ-APC (clone B27), anti-human TNF-α -PE (clone MAB11), anti-IL-2 (clone MQI- 17H12, all from Becton Dickinson), and anti-CD107a-PE (clone H4A3, Pharmingen). Complete peptide sets for SIVmac239 were obtained from the NIH AIDS Research & Reference Reagent Program. In all experiments at least 200,000 T cells were acquired and analyzed.

### Plasma levels of chemokines and cytokines

Plasma levels of the beta-chemokines CCL3/MIP-1α, CCL4/MIP-1β, and CCL5/RANTES in conjunction with other cytokines and chemokines were measured using a sandwich immunoassay-based protein array system, the human cytokine 25-Plex (BioSource International), as instructed by the manufacturer and then read by the Bio-Plex array reader (Bio-Rad Laboratories), which uses fluorescent bead-based technology from Luminex.

## Supporting Information

Text S1 Supplementary figure legends, text, and references.
(0.05 MB DOC)

Table S1 Values for δ and μ for each animal, including lower and upper 95% confidence intervals. Values for δ (half-life of short-lived cells) and μ (half-life of long-lived cells) were estimated based on Eq. 1. 95% confidence intervals were calculated from 500 bootstrap replicates.
(0.02 MB XLS)

Figure S1 Effects of fitting the viral load data with a model that assumes the viral load is in steady state, when in reality viral load is increasing. Surrogate data for SIV kinetics with virus not in steady state (black dots) was created using Eq. 2 (Text S1) with the rate of virion production p allowed to increase as CD8 levels decline in order to account for changes in viremia caused by CD8+ lymphocyte depletion. This data was generated to agree with the change in viremia observed for animal Rsq8. At t = 0, the model assumes combination drug therapy begins with an effectiveness of 99%. The surrogate data was then fit with Eq. 1 and parameters estimated. The best fitting solution is shown by the orange line. The parameters estimated in this way were <3.5% different than the “true” parameters used to generate the data.
(1.69 MB TIF)

Figure S2 CD4+ T cell data used to estimate the change in target cells after CD8+ lymphocyte depletion. Measured CD4+ T cell values for Rsq8 in late chronic infection, (black line) and data smoothed by using a 3 point moving average (purple line). The 3-point moving average was then fit using linear regression to obtain the parameters α and T_0_ used in the supplemental text to define the T cell increase during CD8+ lymphocyte depletion. Analysis of the surrogate SIV RNA data indicates that the effect of changes in CD4+ T-cells and SIV RNA due to CD8+ lymphocyte depletion has a negligible (<3.5%) effect on the estimates of δ and μ when the drug effectiveness is high (∼99%).
(1.97 MB TIF)
