# Supplementary material for: CD8+ Lymphocytes Control Viral Replication in SIVmac239-Infected Rhesus Macaques without Decreasing the Lifespan of Productively Infected Cells
Source: PLoS Pathog. 2010 Jan 29;6(1):e1000747. doi: 10.1371/journal.ppat.1000747 (PMC2813271; doi:10.1371/journal.ppat.1000747)
Supplement: Text S1 — Supplementary figure legends, text, and references. (0.05 MB DOC) [file ppat.1000747.s001.doc]

**Supporting Information**

**Supplementary Figure Legends**

**Supplementary Table 1.** Values for δ and μ for each animal, including lower and upper 95% confidence intervals. Values for δ (half-life of short-lived cells) and μ (half-life of long-lived cells) were estimated based on Eq. 1. 95% confidence intervals were calculated from 500 bootstrap replicates.

**Supplementary Figure 1.** Effects of fitting the viral load data with a model that assumes the viral load is in steady state, when in reality viral load is increasing. Surrogate data for SIV kinetics with virus not in steady state (black dots) was created using Eq. 2 with the rate of virion production *p* allowed to increase as CD8 levels decline in order to account for changes in viremia caused by CD8+ lymphocyte depletion. This data was generated to agree with the change in viremia observed for animal Rsq8. At t=0, the model assumes combination drug therapy begins with an effectiveness of 99%. The surrogate data was then fit with Eq. 1 and parameters estimated. The best fitting solution is shown by the orange line. The parameters estimated in this way were <3.5% different than the “true” parameters used to generate the data.

**Supplementary Figure 2.** CD4+ T cell data used to estimate the change in target cells after CD8+ lymphocyte depletion. Measured CD4+ T cell values for Rsq8 in late chronic infection, (black line) and data smoothed by using a 3 point moving average (purple line). The 3-point moving average was then fit using linear regression to obtain the parameters α and T_0_ used in the supplemental text to define the T cell increase during CD8+ lymphocyte depletion. Analysis of the surrogate SIV RNA data indicates that the effect of changes in CD4+ T-cells and SIV RNA due to CD8+ lymphocyte depletion has a negligible (<3.5%) effect on the estimates of δ and μ when the drug effectiveness is high (~99 %).

**Supplementary Text**

**Supplementary Note 1.** Surrogate data was created to determine the effects of changes in steady-state viremia after CD8+ lymphocyte depletion by simulating the long-lived infected cell model given by equations:

*dT*/dt = (1-ε(t)) k VT_o_-δI* *(2a)*

*dM*/dt = (1-ε(t)) k VM_o_-μM* (2b)*

*dV/dt = N(t)δ I+p(t)M - cV (2c)*

where *T** and *M** are the densities of short-lived and long-lived productively infected cells, respectively, *V* is the virus (SIV RNA) concentration, *T_0_* and *M_0_* are the steady state (baseline) levels of short-lived and long-lived target cells (assumed here to be constant) and *ε*(t) is the effectiveness of ART in blocking reverse transcription. If ε=1 then the therapy is 100% effective and all new infections are blocked. The parameter *k* is the rate of viral infection, *N* is the viral burst size from short-lived infected cells, and *p(t)* is the rate of viral production per long-lived infected cell. As shown in [1] if ε=1 and treatment is started at steady state, then *V(t)* is given by equation *(1)* where *t* is the time on therapy. To simulate the effects of CD8+ lymphocyte depletion, we assume that CD8+ T cells release a factor that acts to decrease viral production. Thus, depleting CD8+ lymphocytes will increase virus production as observed. Other mechanisms could also be used to increase virus production, but here our main interest is simply to study the effect of increased virus before initiation of ART. The first CD8+ lymphocyte-depleting antibody was given 5 days before the start of ART, and by day 8 after the start of ART, CD8+ T cells have reappeared. Thus, choosing *t*=0 as the time therapy is started, we assume that between days -5 and day 8, viral production per infected cell is elevated. For simplicity, this rate was chosen the same for the short and long-lived infected cell populations, i.e. *Nδ = p*, although this need not be the case. Further, we assume that before CD8+ lymphocyte depletion, the system is in steady state and that no antiretroviral drugs are present, i.e. ε=0. At the pre-treatment steady state M_0_= μ (c/(p_s_k)-T_0_/δ) and T*=(KV_s_T_0_)/δ and M* = kV_s_ (c/(p_s_k)-T_0_/δ) , where V_s_, the pre-treatment viral load, was chosen as 9.62 x 10^4^ copies/ml (to represent monkey RSq8’s data in late chronic infection before CD8+ lymphocyte depletion) and T_0_ =10^6^/ml (average of CD4+ T cell count in all monkeys and in both phases before therapy). The other parameters were c=23 day^-1^; *p=p_s_* =100 day^-1^, δ = 1.99 day^-1^, ε =0.99 (during therapy i.e. for *0 ≤ t ≤ 28)*, μ=0.2 day^-1^, *k*=4x10^-7^ ml day^-1^*.* Lastly, we assumed that during CD8+ lymphocyte depletion, i.e. for -5 ≤ t < 8 that *p* was increased and took on values of either 110, 130, 150, or 200 day^-1^. After day 8, *p* was returned to its pre-treatment steady state value p_s_=100 day^-1^. Simulating this modified system of equations, with parameters as given above, we generated sets of surrogate data that were then fitted with equation *(1)* in the text. The surrogate data was sampled every 12 hours for 7 days after initiation of therapy to generate the datasets for fitting (Supplementary Figure 1). For all four values of increased viral production we found that with ε=0.99, δ and μ were both underestimated and within 3.5% of their true values. For example, although δ=1.99 day^-1^ was used to generate the surrogate data, δ was estimated at 1.96 and 1.92 day^-1^, with p=110 and 200 day^-1^, respectively. Similarly, μ=0.2 day^-1^ was used to generate the surrogate data and then was estimated as μ=0.199 and 0.196 day^-1^, with p=110 and 200 day^-1^, respectively.

**Supplementary Note 2.** To determine the impact of increased CD4+ T cell pools as well as increased viremia during CD8+ lymphocyte depletion, we assumed that *T* rather than being held constant at *T_0_* in eqn *(2a)* is variable, i.e., we replaced the term (1-ε)*kVT_0_* by (1-ε)*kVT*, where *T* was given by *dT/dt = α(t)* with α(t)= 0 for *t < 0*, *T(0*)= 275 x10^3^ /ml, and α =27.3 x 10^3^/(ml/day) (*0 ≤ t ≤ 7).* This choice of *T(0)* and α gives rise to a CD4+ T cell increase similar to that observed in monkey Rsq8 (Supplementary Figure 2)*.* Simulating this modified system of equations, with parameters as given above, and *p* varied as above generated a set of surrogate data that was then fit with equation *(1)* in the text. As in the case of fixed target cell levels, for all four values of increased virus production we found that with ε=0.99, δ and μ were again underestimated and within 3.5% of their true values. This is not surprising since with ε close to 1 the impact of changing levels of target cells should be negligible since these levels only enter in the model in terms of the form *(1−ε)kVT*. We thus also studied the effects of assuming lower efficacy of therapy.

Equation *(1)* was derived assuming ε=1(1). When ε <1, and eqn, *(1)* is used to fit data, δ and μ are is always underestimated because in reality more killing of infected cells is needed to counterbalance their continuing generation by de novo infection [2]. Thus, if we generate surrogate data with lower values of ε and fit the surrogate data with equation *(1)* we will obtain lower estimated values of δ and μ, irrespective of whether or not the system is originally in steady state. We also confirmed this by using the procedure given above for non steady-state viral levels and increased numbers of target cells. Thus, these simulations showed that the actual values of δ and μ in systems with CD8+ lymphocyte depletion may be even higher than we estimate. For example, the underestimate of δ caused by CD4+ T cell increase and ε=0.8 was 6.2 % (w/o CD8+ lymphocyte depletion) and 15.6 % (w/ CD8+ lymphocyte depletion, p_L_=200 day^-1^) respectively, while under the same conditions the underestimates of μ were 19 % (w/o CD8+ lymphocyte depletion) and 42 % (w/ CD8+ lymphocyte depletion). This larger underestimate of μ with CD8+ lymphocyte depletion may be the reason that the estimated lifespan of long-lived cells (1/μ) cited in the main text is larger with CD8+ lymphocyte depletion than without it (9.6 vs 8.8 days). However, these underestimates do not change our conclusion in the main text that CD8+ T cells are not required for the rapid loss of productively infected cells.

**Supplementary Note 3.** In order to determine whether the number of animals used were adequate to detect a difference in infected cell lifespan with CD8+ lymphocyte depletion, a power analysis was completed. The average lifespan of short-lived productively infected cells for all treatment periods taken together was 1.0 day, with a standard deviation of 0.3 days, which is consistent with a standard deviation of 0.4 days found in a smaller previous study [3]. If we assume that CD8+ T cells are responsible for the loss of productively infected CD4+ T cells, then we expect that CD8+ lymphocyte depletion would increase the lifespan of these cells to that of an activated (possibly effector) CD4+ T cell of several days. In mice this lifespan has been estimated to be at least ~5 days [4]. Thus, we need to be able to identify an increase in lifespan of 4 days. Given the small standard deviations observed, it is intuitive that we should be able to find such a difference very reliably with our study. Indeed, a priori power calculation indicated that with 10 animals, with a standard deviation of life span of 0.4 days, we should be able to identify in this crossover study a difference of 0.7 days with more than 90% probability. After the study analysis, using Schuirmann’s two one-sided tests to assess equivalence [5, 6] indicates that we can reject the hypothesis that the life spans with and without CD8+ lymphocytes are not equivalent at the 0.05 level, if we define equivalence as the life spans not differing by more than 0.25 days. Thus, these results give us confidence that depletion of CD8+ lymphocytes indeed does not increase substantially the lifespan of productively infected cells.

**Supplementary References**

1. Perelson, A. S., et al. *Nature* **387**, 188 (May 8, 1997).

2. Perelson, A. S., Neumann, U., Markowitz, M., Leonard, J. M., Ho, D. D. *Science (New York, N.Y* **271**, 1582 (Mar 15, 1996).

3. Nowak, M. A., et al., *Journal of virology* **71**, 7518 (Oct, 1997).

4. De Boer, R. J. , Homann, D., Perelson, A. S. *J Immunol* **171**, 3928 (Oct 15, 2003).

5. Schuirmann, D. J. *J Pharmacokinet Biopharm* **15**, 657 (Dec, 1987).

6. Hoenig, J., Heisey, D.M. *The American Statistician* **55**, 19 (2001).
