# Supplementary figures and images for: CD8+ Lymphocytes Control Viral Replication in SIVmac239-Infected Rhesus Macaques without Decreasing the Lifespan of Productively Infected Cells
Source: PLoS Pathog. 2010 Jan 29;6(1):e1000747. doi: 10.1371/journal.ppat.1000747 (PMC2813271; doi:10.1371/journal.ppat.1000747)

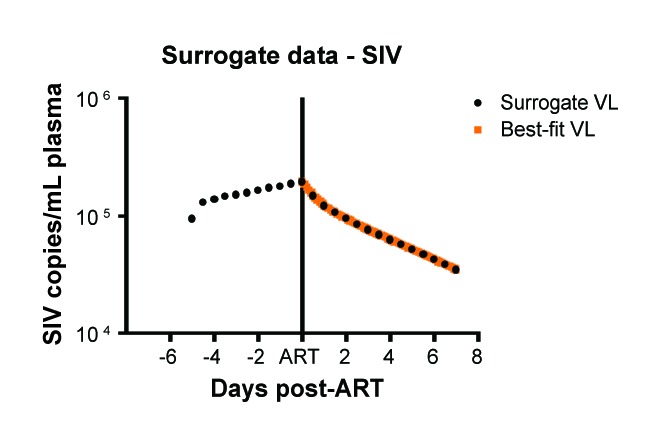

Supplement: Figure S1 — Effects of fitting the viral load data with a model that assumes the viral load is in steady state, when in reality viral load is increasing. Surrogate data for SIV kinetics with virus not in steady state (black dots) was created using Eq. 2 (Text S1) with the rate of virion production p allowed to increase as CD8 levels decline in order to account for changes in viremia caused by CD8+ lymphocyte depletion. This data was generated to agree with the change in viremia observed for animal Rsq8. At t = 0, the model assumes combination drug therapy begins with an effectiveness of 99%. The surrogate data was then fit with Eq. 1 and parameters estimated. The best fitting solution is shown by the orange line. The parameters estimated in this way were <3.5% different than the “true” parameters used to generate the data. (1.69 MB TIF) [file ppat.1000747.s003.tif]

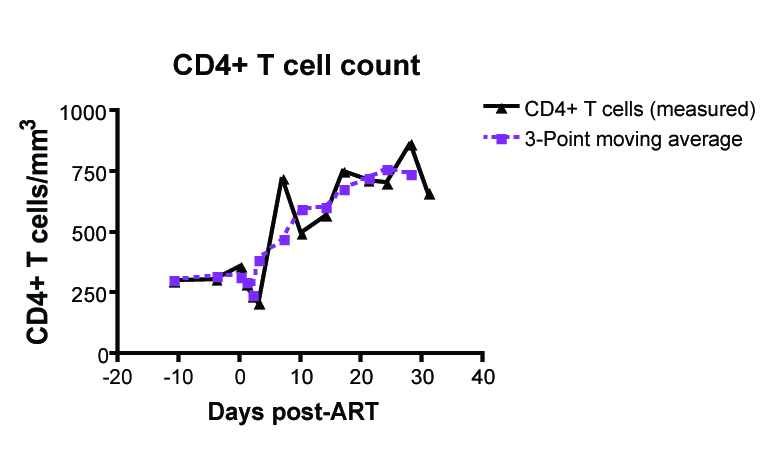

Supplement: Figure S2 — CD4+ T cell data used to estimate the change in target cells after CD8+ lymphocyte depletion. Measured CD4+ T cell values for Rsq8 in late chronic infection, (black line) and data smoothed by using a 3 point moving average (purple line). The 3-point moving average was then fit using linear regression to obtain the parameters α and T0 used in the supplemental text to define the T cell increase during CD8+ lymphocyte depletion. Analysis of the surrogate SIV RNA data indicates that the effect of changes in CD4+ T-cells and SIV RNA due to CD8+ lymphocyte depletion has a negligible (<3.5%) effect on the estimates of δ and μ when the drug effectiveness is high (∼99%). (1.97 MB TIF) [file ppat.1000747.s004.tif]
